# A systematic review of risk and protective factors for depressive symptoms in older adults with visual impairment

**DOI:** 10.1093/geroni/igaf122.4337

**Published:** 2025-12-31

**Authors:** Haruno Suzuki, Heather Leutwyler, Jacqueline Torres, Kenneth Covinsky, Margaret Wallhagen

**Affiliations:** University of California, San Francisco, San Francisco, California, United States; University of California, San Francisco, San Francisco, California, United States; University of California, San Francisco, San Francisco, California, United States; University of California, San Francisco, San Francisco, California, United States; University of California, San Francisco, San Francisco, California, United States

## Abstract

Depressive symptoms are prevalent among older adults with visual impairment (VI), yet there is no synthesis of modifiable factors to support prevention and early treatment programs. This systematic review aimed to identify risk and protective factors associated with depressive symptoms in older adults with VI. The protocol was registered with PROSPERO (CRD42024602520). Eligible studies were quantitative research that examined factors associated with depressive symptoms in adults aged 60 or older with VI. Five electronic databases (PubMed, Web of Science, Embase, PsycINFO, and CINAHL) were searched in November 2024 in collaboration with a medical librarian. Three reviewers independently screened titles, abstracts, and full-texts and assessed methodological quality using the National Heart, Lung, and Blood Institute’s Quality Assessment Tool. Out of 13,761 records identified, 22 studies met the eligibility criteria, including 17 cross-sectional and 5 longitudinal designs. The majority of studies were assessed as “Fair” quality due to a lack of sample size justification and the inability to establish temporality. Common risk factors for depressive symptoms included older age, greater self-reported visual difficulty, poor self-rated health, and greater ADL/IADL limitations. Protective factors included greater social support and use of rehabilitation services. This review suggests that functional limitation may be a key modifiable risk factor associated with depressive symptoms. Our analysis also highlights the critical need for more robust longitudinal studies, standardized definitions of VI, and assessment of cognitive function in future research. Findings may guide clinicians, researchers, and policymakers in developing novel strategies for clinical screening and targeted interventions.

